# Assessment of frontier Large Language Models in sleep medicine

**DOI:** 10.3389/fdgth.2026.1769386

**Published:** 2026-04-29

**Authors:** Anshum Patel, Het Contractor, Hayden Heninger, Sai Krishna Vallamchetla, Pengze Li, Cui Tao, Joseph Cheung

**Affiliations:** 1 Division of Pulmonary, Allergy and Sleep Medicine, Mayo Clinic, Jacksonville, FL, United States; 2 Department of Artificial Intelligence and Informatics, Mayo Clinic, Jacksonville, FL, United States

**Keywords:** artificial intelligence, clinical decision support, diagnostic accuracy, large language models, sleep medicine

## Abstract

**Study objectives:**

To evaluate and compare the performance of two proprietary frontier large language models (LLMs), ChatGPT-5 and Grok-4, on diagnostic reasoning and foundational knowledge tasks within the specialty domain of sleep medicine.

**Methods:**

The models were evaluated on two tasks: case-based reasoning using 79 clinical vignettes from the AASM Case Book of Sleep Medicine and knowledge assessment using 897 multiple-choice questions (MCQs) from board review materials. For vignettes, final diagnosis was scored by concept-level exact match, and differential diagnosis (DDx) was scored on a fixed top-5 output using concept-level matching with synonym normalization to compute precision, recall, and *F*1-score. MCQ performance was the proportion correct. Inter-model performance was compared using the Mann–Whitney *U* test.

**Results:**

Both models achieved high accuracy for final diagnosis (92.4% for both; 95% CI 86.4, 98.4) and MCQs (ChatGPT-5: 93.0%; Grok-4: 92.8%). However, performance on generating a comprehensive differential diagnosis was suboptimal, with modest *F*1-scores for both ChatGPT-5 (0.55 ± 0.20) and Grok-4 (0.59 ± 0.20). There were no statistically significant differences in performance between the two models across any metric (*p* > 0.05).

**Conclusions:**

Frontier LLMs demonstrated high accuracy in sleep medicine tasks requiring knowledge recall and direct pattern recognition but showed more limited performance in complex clinical reasoning tasks such as generating a comprehensive differential diagnosis. These findings suggest that current general-purpose models may be more reliable for focused knowledge support than for broad hypothesis generation. Future studies should evaluate whether domain-adapted models or clinician-in-the-loop workflows can improve real-world diagnostic usefulness and safety.

## Highlights

Widely used proprietary frontier large language models (LLMs) show promise for medical applications, but their diagnostic capabilities in specialized fields like sleep medicine are not well-characterized. This study provides a rigorous evaluation of two leading LLMs on both knowledge-based questions and complex clinical vignettes, uniquely assessing their ability to generate a differential diagnosis. Our findings reveal a critical performance gap: while the models demonstrate expert-level knowledge recall for single correct answers, they show significant limitations in the nuanced clinical reasoning required for differential diagnosis. This evidence strongly supports a physician-AI collaborative model, where AI serves as a powerful knowledge-augmentation tool, thereby enhancing clinical workflow and promoting patient safety in the era of artificial intelligence.

## Introduction

Large language models (LLMs) are generative artificial intelligence (AI) technologies that are already showing impressive capabilities for clinical tasks, but evidence from specialty domains remains mixed. Since 2023, LLMs have been investigated for a wide range of applications in medicine, including clinical decision support, medical research, and professional education (1). These models can streamline administrative workflows by efficiently summarizing complex medical records, demonstrating performance comparable to clinicians but at a significantly faster pace (2). This has spurred widespread investigation into their potential to augment clinical practice, with some studies showing that AI assistance can significantly improve physician diagnostic accuracy in complex cases (3).

A primary area of focus has been the diagnostic capability of LLMs, with several studies benchmarking their performance against physicians. For instance, when measuring diagnostic accuracy, AI performance is comparable to expert-level in some cases (4). Another report highlighted a model's proficiency in resolving a particularly difficult diagnostic case (5). However, this impressive performance has raised critical questions about whether these models are truly reasoning or are engaged in sophisticated pattern recognition (6). Known limitations, such as lack of metacognitive awareness underscore the risks of premature clinical adoption and highlight the need for rigorous, domain-specific evaluations. We compared two general purpose frontier LLMs, ChatGPT-5 and Grok-4, on sleep medicine case vignettes and board-style questions to assess diagnostic capacities and foundational knowledge.

## Methods

We evaluated ChatGPT-5 and Grok-4 across two distinct tasks in sleep medicine: case-based reasoning and multiple-choice knowledge. For case-based reasoning, we utilized 79 clinical vignettes representing ICSD-3-TR disorders, sourced from *AASM Case Book of Sleep Medicine*, 3rd edition (7). The cases were selected to represent a broad spectrum of sleep disorders, including insomnia (*n* = 6), sleep-related breathing disorders (*n* = 26), central disorders of hypersomnolence (*n* = 15), circadian rhythm sleep-wake disorders (*n* = 7), parasomnias (*n* = 10), sleep-related movement disorders (*n* = 9), and other sleep disorders (*n* = 5). For the 29 vignettes that originally contained media-based data (e.g., polysomnography, sleep diaries, or EEG findings), no cases were excluded. Instead, the diagnostic-relevant findings were converted into text by a board-certified sleep physician so that the full case set could be evaluated in a text-only framework. This approach was intended to preserve the key clinical information while minimizing differential bias between models; however, we acknowledge that any physician-mediated conversion of multimodal data into text may introduce subtle interpretive framing. We performed a subgroup analysis comparing these 29 PSG/EEG-text cases with the 50 narrative-only cases.

For each case, models were tasked with standardized prompt to generate (1) a single final diagnosis, and (2) a list of differential diagnoses (DDx). The standardized prompt was:

1. For differential diagnosis- “Based on the provided case details, generate the top 5 differential diagnoses and identify the most likely final diagnosis(es): [Paste Clinical Vignette]”
2. For final diagnosis- “*Based on the provided case details identify the most likely final diagnosis: [Paste Clinical Vignette]*”
3. For MCQ- “*Select the single best answer for given multiple choice question [question stem and all answer choices]*”

For DDx evaluation, the reference standard was the differential diagnosis list provided in the AASM case material. For each case, true positives were defined as the number of unique models DDx entries that matched the reference DDx list; false positives were model-generated DDx entries without a match; and false negatives were reference DDx entries not recovered by the model. Precision was calculated as TP/(TP + FP), recall as TP/(TP + FN), and *F*1-score as 2 × precision × recall/(precision + recall). Because these metrics are sensitive to response length, evaluation was performed on a fixed top-5 DDx output. Final-diagnosis accuracy was measured separately using concept-level exact-match agreement with the reference final diagnosis.

Foundational knowledge was assessed using 897 multiple-choice questions (MCQs) from BoardVitals and *AASM Sleep Qs—Board Review 2.0*. MCQ performance was scored as the proportion correct (8, 9). A standardized prompt was used for all MCQs. We employed a “zero-shot” prompting strategy without iterative refinement or testing of prompt variations. This approach was chosen to simulate a real-world scenario where a layperson or non-expert clinician queries the model using natural language, rather than utilizing specialized “prompt engineering” techniques to artificially maximize performance. To ensure independent trials, the conversation history was cleared before each new vignette was presented, preventing carryover effects. The AASM and BoardVitals are subscription-based resources intended to minimize the likelihood that the specific case content was part of the models' training corpora, thus reducing the risk of data contamination and model overfitting. This study utilized de-identified, published educational material and as no patient health information was involved, Institutional Review Board approval and patient consent were not required. All the responses from models were systematically collected and documented (Supplementary Data S1). Item-level comparisons between models were performed using the Mann–Whitney *U* test, and results are reported as means ± standard deviation (SD) with 95% confidence intervals (CI). All statistical analyses were performed using Python (version 3.9) with the scikit-learn libraries.

## Results

**Figure 1:**
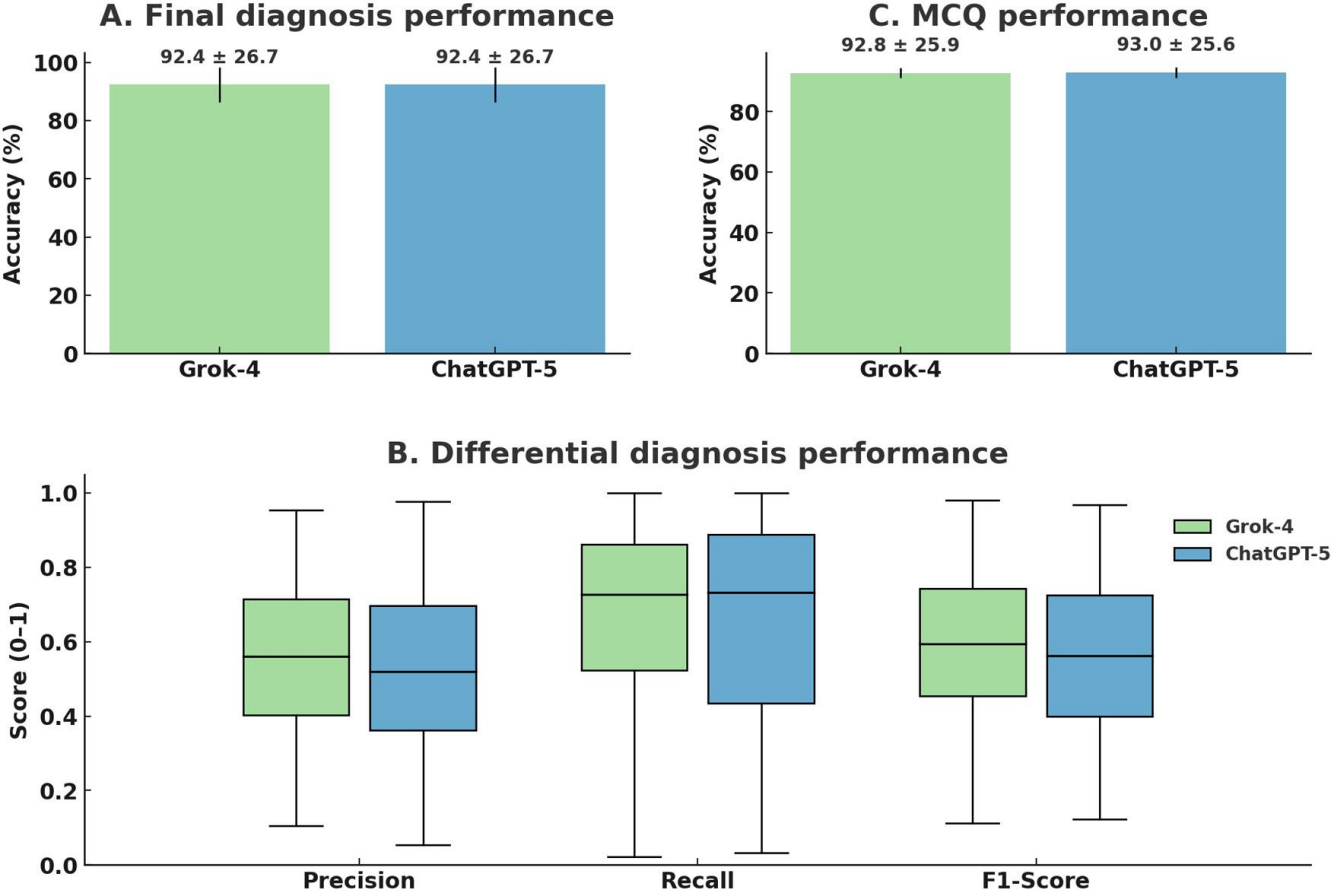
Differential and final diagnostic performance of ChatGPT-5 and Grok 4 on clinical vignettes. Diagnostic performance of ChatGPT-5 and Grok-4 on clinical vignettes and MCQs. **(A)** Bar chart of exact-match final-diagnosis accuracy for each model; values inside bars are percentages. **(B)** Box plots of precision, recall, and F1-score for differential diagnosis across 79 cases; boxes denote IQR with median lines and outliers as points; means are indicated by dots. **(C)** Bar chart of MCQ accuracy across 897 items. No statistically significant model differences were observed for any metric (all *p* > 0.05; Mann–Whitney U test ). *F*1, harmonic mean of precision and recall; SD, standard deviation.

The performance of both models was strong on tasks requiring a single correct answer. In the case-based reasoning task, both ChatGPT-5 and Grok-4 demonstrated high accuracy in identifying the single final diagnosis, correctly identifying the primary condition in 73 out of 79 cases (92.4%; 95% CI 86.4, 98.4) (Table 1; Figure 1A). Similarly, for the MCQ-based knowledge assessment, both models exhibited high accuracy, with ChatGPT-5 correctly answering 93.0% and Grok-4 answering 92.8% of the 897 questions (Figure 1C).

**Table 1:** Performance of Grok-4 and ChatGPT-5 on specialized clinical vignettes and foundational knowledge assessments.

Metric		ChatGPT-5 (mean ± SD) [95% CI]	Grok-4 (mean ± SD) [95% CI]	Mann–Whitney *U*	*p*-value
Differential diagnosis accuracy	Precision	0.50 ± 0.21 (0.46, 0.55)	0.55 ± 0.21 (0.50, 0.59)	3,463.0	0.214
	Recall	0.64 ± 0.26 (0.58, 0.69)	0.68 ± 0.24 (0.62, 0.73)	3,391.0	0.344
	*F*1-score	0.55 ± 0.20 (0.50, 0.59)	0.59 ± 0.20 (0.54, 0.63)	3,466.5	0.228
Final diagnosis accuracy (%)		92.4 ± 26.7 (86.4, 98.4)	92.4 ± 26.6 (86.4, 98.4)	3,120.5	1.000
MCQ accuracy (%)		93.0 ± 25.6 (91.3, 94.7)	92.8 ± 25.9 (91.1, 94.5)	401,407.5	0.855

Performance metrics for the Grok-4 and ChatGPT-5 models across 79 clinical vignettes (final and differential diagnosis accuracy) and 897 multiple-choice questions (MCQ accuracy). Differential diagnosis accuracy is detailed by precision, recall, and *F*1-score. Data is reported as mean ± SD (95% CI). Inter-model comparison utilized the Mann–Whitney *U* test. SD, standard deviation; CI, confidence interval.

In contrast to the high accuracy on single-answer tasks, performance on the more complex clinical reasoning task of generating a comprehensive differential diagnosis was modest. Both models yielded suboptimal *F*1-scores, with Grok-4 (0.59 ± 0.20) performing slightly better than ChatGPT-5 (0.55 ± 0.20). A detailed breakdown of this metric shows Grok-4 also achieved higher mean scores for both precision (0.55 vs. 0.50) and recall (0.68 vs. 0.64) when compared to ChatGPT-5 (Table 1; Figure 1B). Despite these numerical differences, a Mann–Whitney *U* test determined that the performance of ChatGPT-5 and Grok-4 was statistically indistinguishable across all tested metrics (*p* > 0.05 in all cases).

### Stratified analysis by physiological data

We additionally stratified the 79 vignettes by the presence or absence of PSG/EEG-derived information converted into text. In the 29 PSG/EEG-text cases, Grok-4 achieved a final-diagnosis accuracy of 93.1% (27/29), with mean precision 0.56 ± 0.20, recall 0.71 ± 0.26, and *F*1-score 0.61 ± 0.20. In the 50 narrative-only cases, Grok-4 achieved a final-diagnosis accuracy of 92.0% (46/50), with mean precision 0.54 ± 0.22, recall 0.66 ± 0.23, and *F*1-score 0.58 ± 0.19. For ChatGPT-5, final-diagnosis accuracy was 96.6% (28/29) in PSG/EEG-text cases and 90.0% (45/50) in narrative-only cases; mean precision was 0.48 ± 0.19 vs. 0.52 ± 0.22, recall 0.62 ± 0.28 vs. 0.64 ± 0.25, and *F*1-score 0.53 ± 0.20 vs. 0.56 ± 0.20, respectively. No subgroup difference was statistically significant for either model across final diagnosis, precision, recall, or *F*1-score (all *p* > 0.05).

## Discussion

Our evaluation of two frontier LLMs, ChatGPT-5 and Grok-4, reveals a remarkable performance in the domain of sleep medicine. Both models demonstrated high accuracy in tasks requiring knowledge and direct pattern recognition. While both models demonstrated high accuracy in tasks requiring a single correct answer, their ability to generate a comprehensive differential diagnosis remained modest. These models were statistically indistinguishable across all prespecified metrics (*p* > 0.05).

The high accuracy observed in identifying a final diagnosis and answering knowledge-based questions marks a notable advancement over prior LLM generations evaluated in sleep medicine (10, 11). For instance, our previous evaluation of ChatGPT-4 on sleep medicine case vignettes yielded an accuracy of 78.9% for final diagnosis, which rose to 92.4% for ChatGPT-5 in the current study (10). This trend is consistent with prior reports showing that with every new iteration, language models can encode considerable clinical knowledge and perform competitively on board-style—questions (12, 13). However, their capacity to generate a comprehensive differential diagnosis with open-ended clinical reasoning and breadth of hypothesis generation was limited (5, 14). This performance gap suggests that current general-purpose models, while proficient in information recall, have not yet developed the strategic reasoning essential for expert clinical judgment. Particularly within medicine where swift and accurate risk stratification is necessary, generating a robust DDx is crucial for patient safety and avoiding premature diagnostic closure (14). Because our study evaluated only two models at a single time point, these findings do not allow firm conclusions about whether further model scaling is approaching a plateau. Rather, they suggest that, within this benchmark, important gaps remain in open-ended diagnostic reasoning. However, with significant potential for sleep medicine practice and education, LLM-driven AI tools could assist clinicians in diagnostic tasks, summarizing complex information, or drafting preliminary interpretations of sleep study data, thereby potentially streamlining workflows and easing cognitive load (10, 11).

General-purpose large language models (LLMs), trained on broad internet datasets, lack domain-specific data and structured reasoning inherent to expert medical practice (15). Consequently, the developmental trajectory for clinical AI is shifting toward specialization using post-training techniques such as supervised fine-tuning, reinforcement learning from human feedback, and retrieval-augmented generation (RAG), which grounds outputs in real-time, evidence-based sources to reduce hallucinations (16). Future applications will likely move beyond standalone models toward specialized AI agents that coordinate complex workflows, a system particularly valuable in high-acuity environments (17). This evolution reinforces a paradigm of physician-AI collaboration rather than replacement. Evidence supports this model, as one study found AI assistance increased physician diagnostic accuracy in a critical care setting from 27% to 58% (3, 18). Achieving safe deployment requires rigorous clinical validation of these capabilities, necessitating that the clinician's judgment remains the final arbiter in all patient care decisions (5).

This study's strengths include the use of proprietary, subscription-only case vignettes and MCQs to mitigate data contamination, ensuring a more authentic assessment of model generalization. Furthermore, we conducted a clinically relevant evaluation by jointly assessing single answer diagnosis alongside differential diagnosis quality and largescale MCQ accuracy, moving beyond simple correctness. However, the study has some limitations. We relied on text-based datasets rather than dynamic, iterative, and multimodal nature of real-world clinical data; scoring choices and prompt formats can influence results; and model versioning is not fully transparent. Also, while this study establishes a baseline for LLM performance against standardized objective benchmarks, it lacks a human clinician control group. Direct comparison of AI outputs against human decision-making in real-world clinical workflows was beyond the scope of this brief report. These points support cautious interpretation and motivate prospective studies embedded in clinical workflows to establish benefits, risks, and costs.

In conclusion, LLMs showed high accuracy for final diagnosis and board-style knowledge, with more limited differential coverage. The next wave of progress will likely focus on the development of specialized AI tools for domain-specific applications, while rigorously evaluating and ensuring their reliability to deliver better patient care. With careful deployment that emphasizes verification, transparent uncertainty, and domain-specific alignment, these systems could augment clinical practice rather than substitutes for human clinical expertise.

## Data Availability

The data supporting the findings of this study are included in the article and its Supplementary Material. The source educational materials used in this study were derived from proprietary subscription-based resources and are not publicly redistributable. Further inquiries can be directed to the corresponding author.
